# (*E*)-2-Methyl-*N*-[4-(methyl­sulfon­yl)benzyl­idene]aniline

**DOI:** 10.1107/S1600536809047199

**Published:** 2009-11-14

**Authors:** Shao-Song Qian, Hong-You Cui

**Affiliations:** a School of Life Sciences, ShanDong University of Technology, ZiBo 255049, People’s Republic of China; b School of Chemical Engineering, ShanDong University of Technology, ZiBo 255049, People’s Republic of China

## Abstract

Mol­ecules of the title compound, C_15_H_15_NO_2_S, display an *E* configuration with respect to the C=N double bond. The crystal structure is stabilized by weak C—H⋯O hydrogen bonds. The dihedral angle between the two aromatic ring planes is 50.41 (12)°.

## Related literature

For background to Schiff base compounds in coordination chemistry, see: Shao *et al.* (2004 ▶).
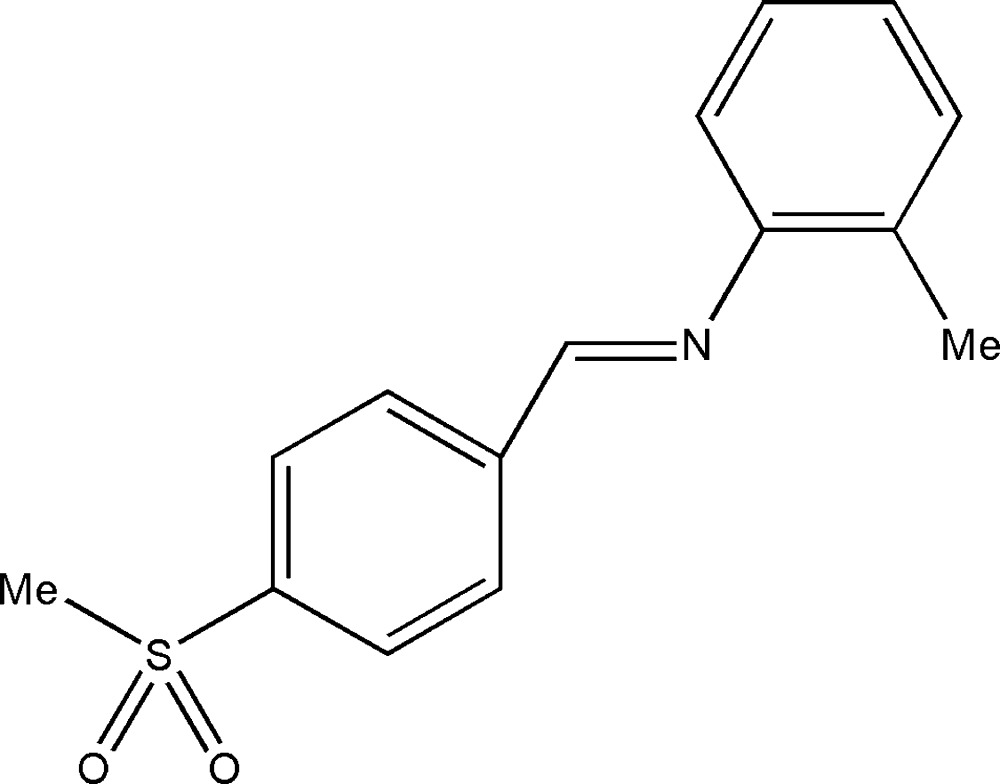


## Experimental

### 

#### Crystal data

C_15_H_15_NO_2_S
*M* *_r_* = 273.34
Monoclinic, 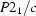

*a* = 11.445 (2) Å
*b* = 7.8770 (16) Å
*c* = 16.132 (3) Å
β = 98.65 (3)°
*V* = 1437.8 (5) Å^3^
*Z* = 4
Mo *K*α radiation
μ = 0.22 mm^−1^
*T* = 293 K
0.30 × 0.20 × 0.20 mm

#### Data collection

Enraf–Nonius CAD-4 diffractometer
Absorption correction: multi-scan (*SADABS*; Sheldrick, 1996 ▶) *T*_min_ = 0.936, *T*_max_ = 0.957
2745 measured reflections
2607 independent reflections
1898 reflections with *I* > 2σ(*I*)
*R*_int_ = 0.026
3 standard reflections every 200 reflections intensity decay: 1%

#### Refinement

*R*[*F* ^2^ > 2σ(*F* ^2^)] = 0.044
*wR*(*F* ^2^) = 0.151
*S* = 1.00
2607 reflections
173 parameters
H-atom parameters constrained
Δρ_max_ = 0.28 e Å^−3^
Δρ_min_ = −0.24 e Å^−3^

### 

Data collection: *CAD-4 Software* (Enraf–Nonius, 1989 ▶); cell refinement: *CAD-4 Software*; data reduction: *CAD-4 Software*; program(s) used to solve structure: *SHELXS97* (Sheldrick, 2008 ▶); program(s) used to refine structure: *SHELXL97* (Sheldrick, 2008 ▶); molecular graphics: *SHELXTL* (Sheldrick, 2008 ▶); software used to prepare material for publication: *SHELXTL* .

## Supplementary Material

Crystal structure: contains datablocks global, I. DOI: 10.1107/S1600536809047199/fj2256sup1.cif

Structure factors: contains datablocks I. DOI: 10.1107/S1600536809047199/fj2256Isup2.hkl

Additional supplementary materials:  crystallographic information; 3D view; checkCIF report

## Figures and Tables

**Table 1:** Hydrogen-bond geometry (Å, °)

*D*—H⋯*A*	*D*—H	H⋯*A*	*D*⋯*A*	*D*—H⋯*A*
C4—H4*A*⋯O1^i^	0.93	2.55	3.274 (3)	135

Symmetry code: (i) 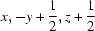.

**Fig. 1.**
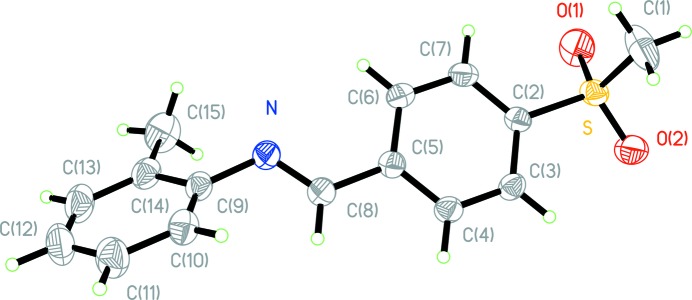
The structure of (I), showing the atom-labelling scheme. Displacement ellipsoids are drawn at the 35% probability level.

## supplementary crystallographic information

### Comment

Schiff base compounds have attracted attention for the development of coordination chemistry related to catalysis and enzymatic reactions, magnetism and molecular archtectures(Shao *et al.*, 2004). As an extension of work on the structural characterization of Schiff base compounds, the crystal structure of the title compound(I), Figure 1, is reported here. The molecule displays a trans-configuration with respect to the C=N double bond. the crystal structure is stabilized by weak C—H···O hydrogen bonds(Figure 2). The dihedral angle between two aromatic ring planes is 50.41 (12)°.

### Experimental

4-(methylsulfonyl)benzaldehyde (0.184g) and o-toluidine (0.107g) were dissolved in acetonitrile (20 ml). The mixture was stirred at room temperature for 10 min to give a clear yellow solution. After keeping the solution in air for 10 d, yellow block-shaped crystals were formed at the bottom of the vessel on slow evaporation of the solvent.

### Refinement

All the H atoms attached to C atoms were placed in geometrical positions and constrained to ride on their parent atoms with C-H distance in the range 0.93-0.98 Å, They were treated as riding atoms, with U_iso_(H) = 1.2U_eq_(C).

### Crystal data

**Table:**

C_15_H_15_NO_2_S	*F*(000) = 576
*M**_r_* = 273.34	*D*_x_ = 1.263 Mg m^−^^3^
Monoclinic, *P*2_1_/*c*	Mo *K*α radiation, λ = 0.71073 Å
Hall symbol: -P 2ybc	Cell parameters from 25 reflections
*a* = 11.445 (2) Å	θ = 9–14°
*b* = 7.8770 (16) Å	µ = 0.22 mm^−^^1^
*c* = 16.132 (3) Å	*T* = 293 K
β = 98.65 (3)°	Block, yellow
*V* = 1437.8 (5) Å^3^	0.30 × 0.20 × 0.20 mm
*Z* = 4	

### Data collection

**Table:**

Enraf–Nonius CAD-4 diffractometer	1898 reflections with *I* > 2σ(*I*)
Radiation source: fine-focus sealed tube	*R*_int_ = 0.026
graphite	θ_max_ = 25.3°, θ_min_ = 1.8°
ω/2θ scans	*h* = 0→13
Absorption correction: multi-scan (*SADABS*; Sheldrick, 1996)	*k* = 0→9
*T*_min_ = 0.936, *T*_max_ = 0.957	*l* = −19→19
2745 measured reflections	3 standard reflections every 200 reflections
2607 independent reflections	intensity decay: 1%

### Refinement

**Table:**

Refinement on *F*^2^	Secondary atom site location: difference Fourier map
Least-squares matrix: full	Hydrogen site location: inferred from neighbouring sites
*R*[*F*^2^ > 2σ(*F*^2^)] = 0.044	H-atom parameters constrained
*wR*(*F*^2^) = 0.151	*w* = 1/[σ^2^(*F*_o_^2^) + (0.1*P*)^2^ + 0.09*P*] where *P* = (*F*_o_^2^ + 2*F*_c_^2^)/3
*S* = 1.00	(Δ/σ)_max_ < 0.001
2607 reflections	Δρ_max_ = 0.28 e Å^−^^3^
173 parameters	Δρ_min_ = −0.24 e Å^−^^3^
0 restraints	Extinction correction: *SHELXTL* (Sheldrick, 2008), Fc^*^=kFc[1+0.001xFc^2^λ^3^/sin(2θ)]^-1/4^
Primary atom site location: structure-invariant direct methods	Extinction coefficient: 0.052 (5)

### Special details

**Table:**

Geometry. All esds (except the esd in the dihedral angle between two l.s. planes) are estimated using the full covariance matrix. The cell esds are taken into account individually in the estimation of esds in distances, angles and torsion angles; correlations between esds in cell parameters are only used when they are defined by crystal symmetry. An approximate (isotropic) treatment of cell esds is used for estimating esds involving l.s. planes.
Refinement. Refinement of F^2^ against ALL reflections. The weighted R-factor wR and goodness of fit S are based on F^2^, conventional R-factors R are based on F, with F set to zero for negative F^2^. The threshold expression of F^2^ > 2sigma(F^2^) is used only for calculating R-factors(gt) etc. and is not relevant to the choice of reflections for refinement. R-factors based on F^2^ are statistically about twice as large as those based on F, and R- factors based on ALL data will be even larger.

### Fractional atomic coordinates and isotropic or equivalent isotropic displacement parameters (Å^2^)

**Table:**

	*x*	*y*	*z*	*U*_iso_*/*U*_eq_	
S1	0.43428 (5)	0.25210 (8)	0.34889 (3)	0.0539 (3)	
N1	0.01690 (16)	0.3139 (2)	0.60356 (12)	0.0527 (5)	
O1	0.35995 (19)	0.2305 (3)	0.27085 (11)	0.0941 (8)	
C1	0.5014 (3)	0.4515 (4)	0.3496 (2)	0.1056 (12)	
H1B	0.5521	0.4550	0.3074	0.158*	
H1C	0.5472	0.4715	0.4036	0.158*	
H1D	0.4417	0.5375	0.3383	0.158*	
C2	0.3445 (2)	0.2644 (3)	0.42882 (13)	0.0447 (5)	
O2	0.52410 (16)	0.1283 (3)	0.37209 (12)	0.0867 (7)	
C3	0.38513 (18)	0.1981 (3)	0.50701 (13)	0.0487 (5)	
H3A	0.4603	0.1507	0.5183	0.058*	
C4	0.31293 (19)	0.2031 (3)	0.56824 (13)	0.0503 (6)	
H4A	0.3397	0.1583	0.6210	0.060*	
C5	0.20111 (19)	0.2740 (3)	0.55184 (13)	0.0463 (5)	
C6	0.1612 (2)	0.3412 (3)	0.47238 (14)	0.0569 (6)	
H6A	0.0865	0.3898	0.4610	0.068*	
C7	0.2329 (2)	0.3353 (3)	0.41107 (14)	0.0569 (6)	
H7A	0.2064	0.3788	0.3580	0.068*	
C8	0.12384 (19)	0.2695 (3)	0.61761 (14)	0.0499 (6)	
H8A	0.1553	0.2328	0.6711	0.060*	
C9	−0.05118 (19)	0.3031 (3)	0.67002 (15)	0.0504 (6)	
C10	−0.0096 (2)	0.3600 (3)	0.75031 (16)	0.0617 (6)	
H10A	0.0659	0.4057	0.7623	0.074*	
C11	−0.0797 (3)	0.3492 (4)	0.81250 (18)	0.0807 (9)	
H11A	−0.0511	0.3865	0.8664	0.097*	
C12	−0.1918 (3)	0.2837 (4)	0.7949 (2)	0.0881 (10)	
H12A	−0.2392	0.2764	0.8367	0.106*	
C13	−0.2333 (2)	0.2291 (4)	0.7156 (2)	0.0805 (9)	
H13A	−0.3090	0.1833	0.7047	0.097*	
C14	−0.1664 (2)	0.2396 (3)	0.65064 (18)	0.0613 (7)	
C15	−0.2132 (3)	0.1814 (4)	0.5632 (2)	0.0894 (9)	
H15A	−0.2931	0.1428	0.5611	0.134*	
H15B	−0.1652	0.0901	0.5478	0.134*	
H15C	−0.2111	0.2741	0.5248	0.134*	

### Atomic displacement parameters (Å^2^)

**Table:**

	*U*^11^	*U*^22^	*U*^33^	*U*^12^	*U*^13^	*U*^23^
S1	0.0647 (4)	0.0564 (4)	0.0429 (4)	0.0082 (3)	0.0156 (3)	0.0032 (2)
N1	0.0492 (11)	0.0500 (11)	0.0603 (12)	0.0039 (9)	0.0122 (9)	0.0041 (9)
O1	0.0922 (14)	0.147 (2)	0.0427 (10)	0.0062 (13)	0.0097 (10)	−0.0103 (11)
C1	0.122 (3)	0.078 (2)	0.135 (3)	−0.0203 (19)	0.078 (2)	−0.001 (2)
C2	0.0524 (12)	0.0402 (11)	0.0421 (11)	0.0007 (9)	0.0093 (9)	0.0013 (9)
O2	0.0940 (13)	0.0980 (16)	0.0764 (12)	0.0453 (12)	0.0395 (11)	0.0257 (11)
C3	0.0436 (11)	0.0587 (13)	0.0426 (11)	0.0065 (10)	0.0019 (9)	−0.0034 (10)
C4	0.0516 (13)	0.0598 (14)	0.0382 (11)	0.0046 (10)	0.0027 (9)	0.0003 (10)
C5	0.0489 (12)	0.0460 (13)	0.0444 (11)	0.0005 (9)	0.0078 (9)	−0.0020 (9)
C6	0.0546 (13)	0.0590 (15)	0.0575 (13)	0.0161 (11)	0.0097 (11)	0.0139 (11)
C7	0.0635 (14)	0.0605 (15)	0.0470 (12)	0.0131 (12)	0.0099 (11)	0.0144 (11)
C8	0.0501 (13)	0.0504 (13)	0.0490 (12)	0.0003 (10)	0.0069 (10)	−0.0014 (10)
C9	0.0466 (12)	0.0421 (11)	0.0642 (14)	0.0031 (9)	0.0140 (10)	0.0026 (10)
C10	0.0569 (13)	0.0595 (15)	0.0708 (15)	0.0010 (12)	0.0159 (11)	−0.0064 (12)
C11	0.087 (2)	0.085 (2)	0.0760 (19)	0.0097 (17)	0.0301 (15)	−0.0102 (15)
C12	0.082 (2)	0.096 (2)	0.098 (2)	0.0081 (18)	0.0495 (19)	0.0055 (19)
C13	0.0546 (16)	0.0719 (19)	0.121 (3)	−0.0029 (13)	0.0317 (17)	0.0098 (18)
C14	0.0500 (13)	0.0496 (14)	0.0848 (18)	0.0007 (11)	0.0124 (12)	0.0043 (12)
C15	0.0661 (17)	0.091 (2)	0.106 (2)	−0.0196 (16)	−0.0010 (16)	−0.0162 (19)

### Geometric parameters (Å, °)

**Table:**

S1—O1	1.420 (2)	C6—H6A	0.9300
S1—O2	1.4257 (18)	C7—H7A	0.9300
S1—C1	1.747 (3)	C8—H8A	0.9300
S1—C2	1.768 (2)	C9—C10	1.385 (3)
N1—C8	1.260 (3)	C9—C14	1.401 (3)
N1—C9	1.420 (3)	C10—C11	1.378 (3)
C1—H1B	0.9600	C10—H10A	0.9300
C1—H1C	0.9600	C11—C12	1.372 (4)
C1—H1D	0.9600	C11—H11A	0.9300
C2—C3	1.380 (3)	C12—C13	1.364 (5)
C2—C7	1.384 (3)	C12—H12A	0.9300
C3—C4	1.380 (3)	C13—C14	1.391 (4)
C3—H3A	0.9300	C13—H13A	0.9300
C4—C5	1.385 (3)	C14—C15	1.503 (4)
C4—H4A	0.9300	C15—H15A	0.9600
C5—C6	1.398 (3)	C15—H15B	0.9600
C5—C8	1.480 (3)	C15—H15C	0.9600
C6—C7	1.377 (3)		
			
O1—S1—O2	117.58 (13)	C6—C7—H7A	120.1
O1—S1—C1	108.53 (17)	C2—C7—H7A	120.1
O2—S1—C1	108.33 (16)	N1—C8—C5	122.2 (2)
O1—S1—C2	108.46 (12)	N1—C8—H8A	118.9
O2—S1—C2	108.73 (10)	C5—C8—H8A	118.9
C1—S1—C2	104.42 (12)	C10—C9—C14	120.2 (2)
C8—N1—C9	118.4 (2)	C10—C9—N1	122.4 (2)
S1—C1—H1B	109.5	C14—C9—N1	117.3 (2)
S1—C1—H1C	109.5	C11—C10—C9	120.3 (2)
H1B—C1—H1C	109.5	C11—C10—H10A	119.8
S1—C1—H1D	109.5	C9—C10—H10A	119.8
H1B—C1—H1D	109.5	C12—C11—C10	120.0 (3)
H1C—C1—H1D	109.5	C12—C11—H11A	120.0
C3—C2—C7	121.0 (2)	C10—C11—H11A	120.0
C3—C2—S1	119.54 (17)	C13—C12—C11	119.7 (3)
C7—C2—S1	119.39 (16)	C13—C12—H12A	120.1
C2—C3—C4	119.2 (2)	C11—C12—H12A	120.1
C2—C3—H3A	120.4	C12—C13—C14	122.2 (3)
C4—C3—H3A	120.4	C12—C13—H13A	118.9
C3—C4—C5	120.7 (2)	C14—C13—H13A	118.9
C3—C4—H4A	119.7	C13—C14—C9	117.4 (3)
C5—C4—H4A	119.7	C13—C14—C15	122.0 (3)
C4—C5—C6	119.5 (2)	C9—C14—C15	120.6 (2)
C4—C5—C8	119.21 (19)	C14—C15—H15A	109.5
C6—C5—C8	121.2 (2)	C14—C15—H15B	109.5
C7—C6—C5	119.9 (2)	H15A—C15—H15B	109.5
C7—C6—H6A	120.1	C14—C15—H15C	109.5
C5—C6—H6A	120.1	H15A—C15—H15C	109.5
C6—C7—C2	119.7 (2)	H15B—C15—H15C	109.5
			
O1—S1—C2—C3	145.99 (19)	C9—N1—C8—C5	179.03 (19)
O2—S1—C2—C3	17.0 (2)	C4—C5—C8—N1	−171.7 (2)
C1—S1—C2—C3	−98.4 (2)	C6—C5—C8—N1	5.3 (3)
O1—S1—C2—C7	−31.9 (2)	C8—N1—C9—C10	45.4 (3)
O2—S1—C2—C7	−160.84 (19)	C8—N1—C9—C14	−137.8 (2)
C1—S1—C2—C7	83.7 (2)	C14—C9—C10—C11	2.2 (4)
C7—C2—C3—C4	0.0 (3)	N1—C9—C10—C11	179.0 (2)
S1—C2—C3—C4	−177.84 (16)	C9—C10—C11—C12	−0.7 (4)
C2—C3—C4—C5	−0.2 (3)	C10—C11—C12—C13	0.1 (5)
C3—C4—C5—C6	0.0 (3)	C11—C12—C13—C14	−1.0 (5)
C3—C4—C5—C8	177.0 (2)	C12—C13—C14—C9	2.4 (4)
C4—C5—C6—C7	0.5 (4)	C12—C13—C14—C15	−179.1 (3)
C8—C5—C6—C7	−176.5 (2)	C10—C9—C14—C13	−3.0 (3)
C5—C6—C7—C2	−0.7 (4)	N1—C9—C14—C13	−179.9 (2)
C3—C2—C7—C6	0.4 (4)	C10—C9—C14—C15	178.5 (2)
S1—C2—C7—C6	178.29 (19)	N1—C9—C14—C15	1.6 (3)

### Hydrogen-bond geometry (Å, °)

**Table:**

*D*—H···*A*	*D*—H	H···*A*	*D*···*A*	*D*—H···*A*
C4—H4A···O1^i^	0.93	2.55	3.274 (3)	135

Symmetry codes: (i) *x*, −*y*+1/2, *z*+1/2.
